# Quantifying cost and health-related quality of life outcomes in different multimorbidity trajectories: a systematic review protocol

**DOI:** 10.1136/bmjopen-2025-102096

**Published:** 2025-08-24

**Authors:** Neave Corcoran, Naomi Wilson, Shona Mackinnon, Anna Blake, Heather Newport, Robert Lawless, Bhautesh Jani, Frances Mair, Rod Taylor, Emma McIntosh

**Affiliations:** 1 General Practice and Primary Care, University of Glasgow School of Health and Wellbeing, Glasgow, Scotland, UK; 2 Health and Wellbeing, University of Glasgow, Glasgow, Scotland, UK; 3 University of Glasgow School of Health and Wellbeing, Glasgow, Scotland, UK; 4 NHS Scotland, Glasgow, Scotland, UK; 5 University of Glasgow, Glasgow, Scotland, UK; 6 MRC/CSO Social and Public Health Sciences Unit & Robertson Centre for Biostatistics, University of Glasgow, Glasgow, Scotland, UK; 7 Health Economics and Health Technology Assessment (HEHTA), University of Glasgow School of Health and Wellbeing, Glasgow, Scotland, UK

**Keywords:** Multimorbidity, Quality of Life, Health Care Costs, Follow-Up Studies, Systematic Review

## Abstract

**Abstract:**

**Introduction:**

Multimorbidity—the presence of multiple long-term conditions (MLTCs)—is a major global public health issue. Most current healthcare systems are not designed for populations with high multimorbidity prevalence. Little work to date has explored the economic burden and health-related quality of life (HRQoL) impacts of MLTCs over time. Health economic evaluations and longitudinal MLTC studies have been highlighted as key multimorbidity research priorities. Understanding incremental healthcare resource use (HCRU), cost and HRQoL impacts as individuals progress along different multimorbidity trajectories is critical to inform the development of future health systems which are both person-centred and adequately resourced for people with MLTCs. This systematic review will synthesise the available evidence on HCRU, costs and HRQoL impacts of different multimorbidity trajectories.

**Methods and analysis:**

The review will be reported according to the Preferred Reporting Items for Systematic reviews and Meta-Analyses 2020 statement. A multi-stranded systematic search strategy will be developed to identify studies of multimorbidity trajectories exploring HCRU and costs (direct and indirect) and/or HRQoL over time. Four databases (Embase, MEDLINE, CINAHL, Web of Science) will be searched; limited to peer-reviewed original, English language, longitudinal, quantitative, adult human studies published on/after 2010. A standard data extraction instrument will be applied to included studies following full-text screening by two reviewers. Studies will be quality-assessed using a tool adapted from the Critical Appraisal Skills Programme cohort studies checklist. Data synthesis will use a Synthesis Without Meta-analysis approach. Outcome measures will include HCRU, healthcare costs and HRQoL changes associated with different multimorbidity trajectories.

**Ethics and dissemination:**

Specific ethics approval has not been sought for this review as it does not directly involve human subjects nor the use of individual-level patient data, and therefore, ethics approval is not required. The review findings will be disseminated via publication in peer-reviewed journal, conference presentations, social media and public engagement events.

**PROSPERO registration number:**

CRD42024537258.

**STRENGTHS AND LIMITATIONS OF THIS STUDY**

- Inclusion of a broad range of multimorbidity, cost and health-related quality of life outcome measures as described here in the review studies will enable systematic capture of relevant evidence.
- The rigorous protocol design is aligned with relevant guidance (Preferred Reporting Items for Systematic reviews and Meta-Analyses Protocols).
- Meta-analysis may or may not be possible depending on the quality of reporting and heterogeneity in included studies.
- Existing evidence in this area may be sparse.

## Introduction

 Multimorbidity—the presence of two or more long-term conditions (LTCs)—is common and increasing and represents a major global public health issue.^1 2^ It is becoming the norm, not the exception, in UK adults and is ‘almost universal’^3^ in older adults; its prevalence increases with age and shows strong socio-economic patterning.^4^

Most current health systems and clinical guidelines are designed around single-condition models of care, unsuited for a population with high multimorbidity prevalence.^5 6^ Major national and international bodies have identified multimorbidity, particularly health economic evaluations of multimorbidity burden, as a research priority.^1^ The National Institute for Health and Care Excellence has stated that the primary care setting is well-suited to managing multimorbidity but identifies challenges to providing optimal management due to current organisational structures, explicitly highlighting the need for high-quality research exploring alternative care models for people with multimorbidity.^5 7 8^

Little work to date has explored the extent and scale of longitudinal costs and impacts on HRQoL as individuals develop multimorbidity over time, progressing from having zero or one single LTC to multiple LTCs (MLTCs), that is, multimorbidity trajectories. The Academy of Medical Sciences reports that “longitudinal data on time trends in costs for the management of multimorbidity are required for a range of settings”.^1^ Longitudinal data on multimorbidity trajectories may refer to, for example, timing or sequencing of LTC accumulation, longitudinal clustering of LTCs or key health-disease state transitions.^9^

Existing studies evaluating healthcare utilisation and direct healthcare costs have consistently demonstrated that multimorbidity is associated with increased primary and secondary healthcare resource utilisation and costs and increased social care costs.^10^,^13^ One common limitation of many studies considering the costs associated with multimorbidity is the scope of costs considered, where non-medical and indirect costs (eg, work absence, loss of income) are often not included, potentially underestimating the true total societal costs and impacts of multimorbidity.^14^ Different combinations (clusters) of specific multimorbid LTCs are associated with differential levels of healthcare usage and cost, for example, combinations of physical and mental health LTCs typically being more costly than multiple physical LTCs.^10 14 15^

A 2022 systematic review by Tran *et al* exploring costs of multimorbidity detected certain time points or ‘transition stages’ in a health trajectory where costs concentrated: around the time of a new diagnosis and in 6 months preceding death.^14^ The authors underscore the importance of further longitudinal multimorbidity cost research to better understand the dynamic nature of health states,^12^ disease trajectories and associated costs, and thereby target future interventions to best prevent multimorbidity progression.^14^

In addition to healthcare resource use (HCRU) and costs, health-related quality of life (HRQoL) forms another key component of many health economic evaluations. There is growing cross-sectional evidence on the inverse relationship between greater LTC counts and HRQoL outcomes across multiple systematic reviews.^16^,^18^ However, key reviews of the association between multimorbidity and HRQoL outcomes have identified a sparsity of longitudinal studies and suggest that longitudinal research is required to aid understanding of the impacts of change in the extent of multimorbidity over time on HRQoL.^16 19^ Research in this area has highlighted significant heterogeneity in studies exploring associations between multimorbidity and HRQoL outcomes.

New research assessing HRQoL impacts associated with different MLTC clusters is also emerging, and recently published work by Steell *et al* has highlighted that MLTC clusters including painful conditions, depression and cardiometabolic disease are consistently associated with substantial deficits in HRQoL.^20^ This work further emphasises an urgent need for future research studying the impacts of longitudinal evolution of MLTC clusters over time (ie, MLTC trajectories) on health-related outcomes such as cost and HRQoL.

Zhang *et al* used a group-based multi-trajectory modelling approach to identify groups of individuals in China following similar multimorbidity trajectories based on accrual of patterned clinical conditions and found that all multimorbidity trajectory groups had higher healthcare utilisation and expenditure as compared with participants who did not develop multimorbidity and furthermore found that different clinically defined multimorbidity trajectory groups used healthcare resources differentially and incurred different health expenditures.^21^

Studies including those by Munyombwe *et al*^22^ and Li *et al*^23^ have explored longitudinal changes in HRQoL related to cross-sectional multimorbidity patterns. Both of these studies found an association between the extent of multimorbidity and steeper decrements in HRQoL over time. Li *et al* also highlight the lack of existing research on prospective, longitudinal associations between multimorbidity and HRQoL.

There remains scarce evidence to date exploring the varying impacts of different patterns of multimorbidity development over time on HRQoL outcomes, and there is a need for further work in this area. There are further limitations in economic evaluation evidence generated due to the varying use of preferred, ‘preference-based’, HRQoL instruments.^24 25^

This systematic review will, for the first time, synthesise the available evidence on HCRU, cost and HRQoL impacts of different trajectories of multimorbidity development.

### Aims/objectives

This review aims to identify studies exploring multimorbidity trajectories and the associated impacts on HCRU, direct and indirect healthcare costs and/or HRQoL outcomes, and synthesise the findings of these studies to answer the following research questions:

What is the impact of different multimorbidity trajectories on HCRU/cost and HRQoL outcomes?

What methods have been used to explore the associations between different multimorbidity trajectories and HCRU/cost and HRQoL?

## Methods and analysis

The review protocol is reported according to the Preferred Reporting Items for Systematic review and Meta-Analyses Protocols (PRISMA-P) 2015 statement.^26^ Early scoping of the literature suggests that included studies may not be amenable to meta-analysis due to significant heterogeneity; therefore, the most appropriate method for data synthesis will be decided at the time of data synthesis and may use Synthesis Without Meta-analysis guidelines.^27^

This review has been registered with PROSPERO, the international registry for systematic reviews (registration number: CRD42024537258).

### Eligibility criteria

Inclusion (and exclusion) criteria for this review are summarised in table 1 according to the Population, Experiment, Comparator, Outcome, Study Design framework^28^ adaptation of Population, Intervention, Comparison, and Outcome methodology^29^ and described further below.

**Table 1:** Review inclusion criteria

PECOS component	Description
Population	Adults (≥18 years old) with or without multimorbidity at baseline who are followed up for their MLTC status
Exposure	Development of different multimorbidity trajectories over time
Comparator	Comparator may be adults with zero or one long-term condition, or comparing one multimorbidity trajectory with another; however, studies that do not include a comparator or control group will not be excluded.
Outcomes	Measure of healthcare resource use and/or direct and indirect costs reported cross-sectionally or longitudinally Measure of health-related quality of life outcomes reported cross-sectionally or longitudinally
Setting	Any healthcare setting
Study design	Longitudinal, quantitative studies
Other exclusions	Ineligible study designs include reviews, systematic reviews, meta-analyses, cross-sectional studies, studies reporting population trends in multimorbidity status as opposed to following a cohort of individual participants longitudinally, case reports, expert opinion/committee reports, editorial pieces/viewpoints, qualitative studies, intervention studies, RCTs, conference abstracts and study protocols.

PECOS, Population, Experiment, Comparator, Outcome, Study Design; RCT, randomised controlled trial.

### Population

The population of interest for this review will be adults (≥18 years old) with or without multimorbidity (two or more LTCs) at baseline, who develop multimorbidity and for whom longitudinal HCRU and/or cost and/or HRQoL data are recorded. This could include people with no LTCs, with a single LTC or with established multimorbidity present at baseline.

### Exposure

The ‘exposure’ of interest is development of different multimorbidity trajectories. To be included, studies must use a measure that explicitly quantifies multimorbidity and may include simple count-based measures of numbers of LTCs, or specifically developed multimorbidity indexes, for example, Charlson Comorbidity Index^30^ and Elixhauser index.^31^

It is expected that there may be significant variability in the way multimorbidity is defined within included studies,^32^ including in terms of which specific LTCs are included.^33^ Based on existing evidence, the inclusion of diagnoses which may be variously considered as conditions or risk factors, such a hypertension, hypercholesterolaemia, obesity, within different multimorbidity measures, is expected to vary.^32^

LTCs will be considered according to the definitions established by Barnett *et al* where closely related conditions such as coronary heart disease and peripheral artery disease are considered as distinct LTCs.^4^ Obesity and high cholesterol will generally be regarded as risk factors rather than LTCs, and hypertension as an LTC, in accordance with Ho *et al*’s 2022 Delphi study.^32^ However, since it is expected that definitions of LTCs and lists of included LTCs will likely vary between included studies, provided that rationale for a given approach is demonstrated, studies will not be excluded on the basis of varying LTC inclusion, provided they meet the other review eligibility criteria.

It is anticipated that there will be substantial variety in the way that ‘multimorbidity trajectories’ is operationalised in studies included in the review.^9^ For example, approaches could focus on disease sequence, that is, the order in which LTCs develop in an individual^34^; timing or rate of multimorbidity development, including age at onset,^35^ longitudinal clusters or groupings of LTCs, which tend to pattern together over time with physical-mental health clusters or cardiometabolic clusters^36^; disease state transitions^37^; and changes in simple LTC count or comorbidity index measure over time.^38^

Depending on the variety of approaches taken to define multimorbidity trajectories found in included studies, data analysis and synthesis methods will be tailored accordingly, for example, by grouping studies that employ similar methodologies for synthesis and presentation of results.

### Comparator

Studies will be eligible for inclusion if they examine trajectories of multimorbidity development over time/longitudinally and the impact of these development trajectories on HCRU, cost and/or HRQoL outcomes. Reporting of multimorbidity trajectories may be by various methodological approaches, for example, multiple linear regression models, sequence analysis, disease state transitions and cluster or group-based approaches.^9^

For evaluating the association between multimorbidity trajectories and HCRU/cost or HRQoL outcomes, studies should report the association between multimorbidity trajectory and the outcome of interest.

Participants who develop multimorbidity may be compared with adults who do not develop multimorbidity during the follow-up period, that is, those with zero or one long-term condition throughout follow-up or may compare the effect size of different multimorbidity trajectories on HCRU/cost or HRQoL outcomes against each other, although studies will not be excluded if there is no comparator.

### Outcomes

Outcome measures in this review will include either or both HCRU and/or costs (including direct and indirect costs, eg, work loss, reduced productivity) or HRQoL changes associated with different multimorbidity trajectories. Specific outcomes of interest are detailed in table 2.

**Table 2:** Outcomes of interest

Outcome domain	Outcomes
HCRU/cost	HCRU Hospitalisations/inpatient care Outpatient Primary care Social care Prescriptions Over-the-counter costs Personal expenditure Private HCRU Other resource use Healthcare costs (where monetary cost is reported alone without detail of resource use) Indirect costs Non-medical costs (transport, meals, accommodation, etc) Loss of income Job loss Reduced productivity Loss of benefits Opportunity costs Perspective Currency Unit cost source(s) Base year for costs Discounting applied Y/N and if Y, discount rate Full economic evaluation Y/N and if Y, economic evaluation framework: cost-effectiveness analysis, cost-utility analysis, cost-benefit analysis, cost-consequence analysis
HRQoL	Validated measures of generic or disease-specific HRQoL, to include preference or non-preference-based measures; mapping algorithm for non-preference-based measures

HCRU, healthcare resource use; HRQoL, health-related quality of life.

### Setting

Studies conducted in any healthcare setting will be eligible, including community, hospital outpatient or inpatient and long-term care settings.

### Study design

We will identify primary longitudinal quantitative prospective or retrospective research studies, to include observational (eg,cohort study) designs, and those which consider ‘natural trajectories’ of multimorbidity development. This may include studies of index long-term conditions with the development of additional long-term conditions—that is, comorbidity studies—where longitudinal development of multimorbidity is reported. Comorbidity studies where longitudinal development of multimorbidity is not reported will be excluded.

### Study identification

#### Electronic searches

A multi-stranded systematic search strategy will be developed in consultation with a university information specialist (librarian), using a combination of Medical Subject Heading terms and keywords to search, and incorporating concepts relating to multimorbidity, trajectories, HCRU and costs, and HRQoL. The search will focus on longitudinal quantitative studies of multimorbidity trajectories and associated HCRU, direct and indirect costs and/or HRQoL impacts.

The search will be performed in four databases—Embase, Medline, CINAHL and Web of Science—and will be limited to peer-reviewed original longitudinal quantitative studies published on or after 2010, in the English language and concerned with adult (≥18 years) human subjects’ development of multimorbidity over time. The start date of 2010 was chosen as the concept of multimorbidity as referred to in this review was defined and developed from the early 2010s.^39 40^ The search will be first conducted in Embase before being adapted to the search syntax conventions of each of the other named databases, and the search will be performed in each database. Embase search strategy is included in online supplemental appendix A, and complete search strategies for each database will be shared via PROSPERO on publication of the completed review.

Electronic searches will be supplemented by hand-searching of reference lists of relevant articles.

### Data collection and analysis

#### Study selection

Studies retrieved through database searches will be exported via EndNote reference manager to DistillerSR systematic reviewing software, and results will be deduplicated. According to the defined inclusion and exclusion criteria, two independent reviewers will screen the title and abstract for each identified study. Any conflicts in inclusion decisions between reviewers will be resolved by discussion, and if consensus cannot be reached, studies will be included for full-text screening.

Full-text screening of potentially eligible studies will be performed by two independent reviewers, and reasons for study exclusion at full-text stage will be recorded. Any conflicts at the full-text screening stage will be resolved by discussion and will include a third reviewer if required.

#### Data extraction

A standard data extraction form will be designed, piloted and applied to those studies included after full-text screening. Data extraction will be performed by two reviewers and will (where available) include items as listed in table 3.

**Table 3:** Data extraction

Domain	Data items
Article identifiers	Authors Title Publication year Journal
Study characteristics	Country World Bank income status of country of study Data source Setting Study design Sample size Sampling/recruitment approach Sex: proportion female Age (mean, range) Ethnicity Socio-economic status Mean income Education Smoking Alcohol BMI Physical activity Follow-up duration Number of data collection points Frequency of data collection Statistical analysis Covariates adjusted for in multivariate analysis Subgroup analyses Additional specific measures Approach to patient/public engagement
Multimorbidity measures	Multimorbidity or comorbidity study Multimorbidity measure type Definition of multimorbidity Included LTCs Multimorbidity status at study baseline Clustering Trajectory measure
HCRU/cost outcomes, informed by the Consolidated Health Economic Evaluation Reporting Standards 2022 (CHEERS 2022)^48^	HCRU Hospitalisations/inpatient care Outpatient Primary care Social care Prescriptions Over-the-counter costs Personal expenditure Private HCRU Other resource use Healthcare costs (where monetary cost is reported alone without detail of resource use) Indirect costs Non-medical costs (transport, meals, accommodation, etc) Loss of income Job loss Reduced productivity Loss of benefits Opportunity costs Perspective Currency Unit cost source(s) Base year for costs Discounting applied Y/N, and if Y, discount rate Full economic evaluation Y/N, and if Y, economic evaluation framework: cost-effectiveness analysis, cost-utility analysis, cost-benefit analysis, cost-consequence analysis
HRQoL outcomes	Validated measures of generic or disease-specific HRQoL, eg, EQ5D-3L EQ5D-5L SF6D SF-12 SF-36 ICECAP HUI SGRQ QALY DALY Other: preference or non-preference-based measure used Use of mapping algorithm for non-preference-based measure Disease-specific instrument used
Results, conclusions and quality	Study key findings/conclusions Quality assessment Limitations

DALY, quality-adjusted life year; EQ5D-3L, three level EuroQol-three dimensional; EQ5D-5L, five level EuroQol-five dimensional; HCRU, healthcare resourse use; HRQoL, health-related quality of life; HRQoL, health-related quality of life; HUI, Health Utility Index; ICECAP, ICEpop CAPability; LTCs, long-term conditions; QALY, disability-adjusted life year; SF-12, 12-item short form health survey; SF-36, 36-item short form health survey; SGRQ, St. George's Respiratory Questionnaire.

#### Quality assessment

Studies fulfilling inclusion criteria will be quality-assessed by two independent reviewers for study quality and risk of bias, using a specifically designed tool outlined in box 1, based on the Critical Appraisal Skills Programme Checklist for cohort studies,^41^ and the Joanna Briggs Institute Checklist for cohort studies,^42^ and adapted according to the research question. Further quality appraisal elements specific to cost of illness studies^43^ and health state utility value studies^44^ have also been incorporated into the tool. Any discrepancies between reviewers will be resolved by consensus or, if required, by consulting a third reviewer.

Box 1 **Quality assessment domains (adapted from Critical Appraisal Skills Programme Checklist for Cohort Studies and Joanna Briggs Institute Cohort Studies Checklist, incorporating cost of illness and health state utility value elements):**

Study details.
Did the study address a clearly focused issue?
Was the cohort recruited in an acceptable way? Were groups similar and recruited from the same population?
Was the exposure accurately measured to minimise bias, that is, measured similarly in both groups, measured in a valid and reliable way?
(a) Was the outcome accurately measured to minimise bias, that is, measured in a valid and reliable way? (b) Where HCRU/cost outcomes are reported, are all included components of resource use identified, measured and valued in monetary terms? Are future costs discounted? Are results presented by cost category/sector? (c) Where HRQoL outcomes are reported, is the measure used valid in the population studied?
(a) Have the authors identified all important confounding factors? (b) Have they taken account of the confounding factors in the design and/or analysis? Were strategies to deal with confounding factors stated?
(a) Was the follow-up of subjects complete enough? And if not, were the reasons for loss to follow-up described and explored? (b) Was the follow-up of subjects long enough? Was follow-up time reported and sufficient to be long enough for outcomes to occur?
What are the results of this study?
How precise are the results?
Do you believe the results?
Can the results be applied to the local population?
Do the results of this study fit with other available evidence?
What are the implications of this study for practice?

### Data synthesis

The appropriate method of data synthesis will be determined after the evaluation of the included studies. Initial scoping of the literature suggests there may be limited existing published evidence addressing our stated research questions and that significant methodological heterogeneity may be found between studies, including in terms of clinical focus, study designs, populations and outcomes of interest, effect measures and statistical methodologies.^45^ For these reasons, meta-analysis may not be possible; therefore, an approach such as Synthesis Without Meta-analysis (SWiM)^27^ may be most appropriate. The SWiM guidelines acknowledge that formal statistical investigation of heterogeneity may not always be possible or appropriate and offer alternative approaches to examine heterogeneity such as ordering tables and/or figures by study characteristics such as study design, population or outcomes. Rationale will be provided for synthesis methods and presentation of study findings, in accordance with PRISMA 2020 statement reporting guidelines.^46^

We will report multimorbidity trajectories where multimorbidity status has been measured longitudinally, which may be by simple count of the number of long-term conditions or according to weighted indices or measures, for example, Charlson Comorbidity Index,^30^ or by some novel or created measure. Descriptive statistics will be presented in table format. Studies will likely include a variety of measures of the effect including HRs, relative risks and ORs. Economic data will include mean cost values and associated confidence intervals, skewness, and . HRQoL data will include scores between zero and one (some negative scores) and CIs.

Confidence in the cumulative quality of the evidence presented in this review will be evaluated using Grading of Recommendations Assessment, Development and Evaluation framework criteria.^47^

### Patient and public involvement

This systematic review will form part of NC’s doctoral thesis. The NHS Research Scotland Primary Care Patient and Public Involvement (PPI) group was consulted in the planning and design of this review protocol from early stages, with additional input from an allocated PPI mentor, PL, on the review as well as the doctoral project as a whole. PPI engagement contributed particularly to the development of the research questions, the selection of relevant outcomes, particularly inclusion of indirect costs, and the use of a double-stranded search strategy encompassing both HCRU/cost and HRQoL outcomes.

Further PPI consultation will be planned regarding dissemination of the review results and public engagement with the review findings.

## Discussion

This systematic review will synthesise the available evidence on HCRU and costs, and HRQoL impacts of different longitudinal trajectories of multimorbidity development.

As multimorbidity becomes the norm rather than the exception in adulthood and older age, healthcare and social care services, as well as policy, need to be designed around the needs of populations with high multimorbidity prevalence.^1 5^ An essential aspect of this is to consider longitudinal aspects of the process by which people accrue multiple long-term conditions through their lives and to identify key inflection points in individuals’ health trajectories where timely intervention may prevent or slow multimorbidity progression.^9^ The timing of interventions is likely to influence costs and HRQoL trajectories, and hence, longitudinal information on HCRU, costs and HRQoL is needed.^20^

There is a small and growing body of longitudinal multimorbidity research, mostly published in recent years. Existing research on multimorbidity trajectories has highlighted considerable heterogeneity in methodologies used, including in study design, population(s) of interest, sampling strategies and analytic approaches,^9^ which may limit the comparability of studies included in this review, as well as wider generalisability of findings.

To our knowledge, this systematic review will be the first to synthesise the evidence on the impact of multimorbidity trajectories on HCRU, cost and HRQoL outcomes. The broad scope of multimorbidity measures and definitions included in this review will enable exploration of the wide range of approaches, which have been used to quantify multimorbidity trajectories to date, particularly in their associations with key HCRU, cost and HRQoL outcomes. The comprehensive search strategy proposed will minimise the chance of missing relevant studies. 2010 has been chosen as an earlier date search limit, based on research from 2013 developing the definition of multimorbidity as conceptualised in this review^39^; therefore, little relevant evidence would be expected to have been published prior to 2010. However, it is possible that this approach may exclude relevant studies published before 2010, and this is acknowledged as a potential limitation. This review will focus on multimorbidity trajectories and therefore may exclude comorbidity studies of index conditions, where development of multimorbidity is not reported.

Early scoping of the evidence base suggests there may be few studies that specifically address the question of interest and that these studies may be heterogeneous in their design, execution and analysis. Given this potential heterogeneity, the most appropriate data synthesis method will be determined once included studies are evaluated. Should the included studies not be amenable to meta-analysis, this will be recognised as a limitation of the review, and SWiM methodology will provide an alternative systematic, transparent and rigorous synthesis approach.^27^

This work is novel, as to date, there is no systematic overview of literature relating to HCRU, cost and HRQoL impacts of different multimorbidity trajectories. As such, this research will likely identify important evidence gaps and therefore avenues for necessary future work exploring, for example, economic evaluation of interventions along the course of multimorbidity trajectories. This research will evidence the wider human consequences for individuals, the health sector and society in terms of HRQoL impacts of multimorbidity trajectories over time.

This systematic review will synthesise evidence in this important, under-researched area of cost and HRQoL impacts of multimorbidity trajectories, providing essential context to optimise future healthcare systems for people with MLTCs and informing novel multimorbidity trajectories research.

Findings from this work could inform health policy, allocation of healthcare resources and guide the design of future health services and interventions which address both cost-effectiveness, optimisation and sustainability concerns, while mitigating HRQoL detriment for people with multimorbidity.

## Ethics and dissemination

Specific ethics approval has not been sought for this review as it does not directly involve human subjects nor the use of individual-level patient data, and therefore, ethics approval is not required.

The review findings will be disseminated via publication in peer-reviewed journals, conference presentations, social media and public engagement events.

## Supplementary material

10.1136/bmjopen-2025-102096 online supplemental file 1
