# Supplementary material for: Quantifying cost and health-related quality of life outcomes in different multimorbidity trajectories: a systematic review protocol
Source: BMJ Open. 2025 Aug 24;15(8):e102096. doi: 10.1136/bmjopen-2025-102096 (PMC12382564; doi:10.1136/bmjopen-2025-102096)
Supplement: online supplemental file 1 [file bmjopen-15-8-s001.pdf]

Database:
Embase 1947-Present, updated daily

#	Query	Results from 23 Jan 2024
1	(Comorbidity or comorbid* or co-morbid*).mp. [mp=title, abstract, heading word, drug trade name, original title, device manufacturer, drug manufacturer, device trade name, keyword heading word, floating subheading word, candidate term word]	636,596
2	(Multimorbidity or multimorbid* or multi-morbid*).mp. [mp=title, abstract, heading word, drug trade name, original title, device manufacturer, drug manufacturer, device trade name, keyword heading word, floating subheading word, candidate term word]	14,479
3	(Multidisease or multi-disease*).mp. [mp=title, abstract, heading word, drug trade name, original title, device manufacturer, drug manufacturer, device trade name, keyword heading word, floating subheading word, candidate term word]	476
4	(Multi* adj2 (disease* or condition* or syndrom* or disorder*)).mp. [mp=title, abstract, heading word, drug trade name, original title, device manufacturer, drug manufacturer, device trade name, keyword heading word, floating subheading word, candidate term word]	175,841
5	multiple chronic conditions/	8,883
6	or/1-5	805,439
7	exp health care cost/	350,036
8	funding/	81,478
9	cost*.ti.	201,491
10	(economic* or pharmaco?economic*).ti.	81,161
11	(price* or pricing*).ti,ab.	76,866

12	(cost* adj2 (effective* or utilit* or benefit* or minimi* or unit* or estimat* or variable*)).ab.	296,701
13	(value adj2 (money or monetary)).ti,ab.	4,265
14	(expenditure? adj3 (health or direct or indirect)).tw.	14,799
15	Health resource utili?ation.mp.	1,368
16	Healthcare utili?ation.mp.	15,664
17	Healthcare expenditure*.mp.	4,646
18	or/7-17	866,253
19	quality adjusted life year.mp. [mp=title, abstract, heading word, drug trade name, original title, device manufacturer, drug manufacturer, device trade name, keyword heading word, floating subheading word, candidate term word]	38,155
20	"quality of life index"/	3,246
21	short form 12/ or short form 20/ or short form 36/ or short form 8/	50,948
22	sickness impact profile.mp. [mp=title, abstract, heading word, drug trade name, original title, device manufacturer, drug manufacturer, device trade name, keyword heading word, floating subheading word, candidate term word]	3,402
23	(quality adj2 (wellbeing or well-being)).ti,ab.	3,992
24	sickness impact profile.ti,ab.	1,250
25	disability adjusted life.ti,ab.	6,832
26	(qal* or qtime* or qwb* or daly*).ti,ab.	35,325
27	(euroqol* or eq5d* or eq 5d*).ti,ab.	31,504
28	(qol* or hql* or hqol* or h qol* or hrqol* or hr qol*).ti,ab.	137,069
29	(health utility* or utility score* or disutilit*).ti,ab.	6,799
30	(hui or hui1 or hui2 or hui3).ti,ab.	3,238
31	health* year* equivalent*.ti,ab.	41
32	(hye or hyes).ti,ab.	185
33	rosser.ti,ab.	143

34	(willingness to pay or time tradeoff or time trade off or tto or standard gamble*).ti,ab.	17,548
35	(sf36 or sf 36 or short form 36 or shortform 36 or shortform36).ti,ab.	50,815
36	(sf20 or sf 20 or short form 20 or shortform 20 or shortform20).ti,ab.	606
37	(sf12 or sf 12 or short form 12 or shortform 12 or shortform12).ti,ab.	12,576
38	(sf8 or sf 8 or short form 8 or shortform 8 or shortform8).ti,ab.	1,218
39	(sf6 or sf 6 or short form 6 or shortform 6 or shortform6).ti,ab.	2,926
40	(RAND 36 or RAND 20 or RAND 12 or RAND 8 or RAND 6).ti,ab.	2,115
41	(self rat* adj2 health).mp.	12,503
42	capability wellbeing.mp.	35
43	ICECAP.mp.	292
44	Ascot instrument.mp.	0
45	(YFC or years of full capability).ti,ab.	37
46	(YSC or years of sufficient capability).ti,ab.	187
47	or/19-46	289,515
48	Case control study.mp. [mp=title, abstract, heading word, drug trade name, original title, device manufacturer, drug manufacturer, device trade name, keyword heading word, floating subheading word, candidate term word]	263,463
49	Longitudinal study/	206,209
50	Retrospective study/	1,565,166
51	Prospective study/	906,544
52	Cohort analysis/	1,108,630
53	(Cohort adj (study or studies)).mp.	497,736
54	(Case control adj (study or studies)).tw.	172,740
55	(follow up adj (study or studies)).tw.	80,532
56	(observational adj (study or studies)).tw.	266,959
57	(epidemiologic$ adj (study or studies)).tw.	128,137
58	trajector*.tw.	134,704
59	longitudinal*.tw.	495,009

60	sequenc*.tw.	1,755,865
61	or/48-60	5,659,290
62	61 not (cross-sectional study/ or 'cross-section*'.mp. or 'prevalence study'.mp.) [mp=title, abstract, heading word, drug trade name, original title, device manufacturer, drug manufacturer, device trade name, keyword heading word, floating subheading word, candidate term word]	5,424,918
63	62 not systematic review/	5,343,559
64	63 not (dna or cell* or bacter* or gene or genes or covid*).mp.	3,058,413
65	6 and 18 and 64	12,380
66	6 and 47 and 64	6,384
67	65 or 66	18,166
68	animal/	2,149,416
69	human/	27,305,967
70	68 not (68 and 69)	1,629,720
71	67 not 70	18,164
72	limit 71 to (adult <18 to 64 years> or aged <65+ years>)	15,242
73	limit 72 to english language	15,097
74	limit 73 to yr="2010 -Current"	14,152
75	limit 74 to (article or article in press)	8,799
