# Supplementary material for: Quantifying cost and health-related quality of life outcomes in different multimorbidity trajectories: a systematic review protocol
Source: BMJ Open. 2025 Aug 24;15(8):e102096. doi: 10.1136/bmjopen-2025-102096 (PMC12382564; doi:10.1136/bmjopen-2025-102096)
Supplement: Supplementary file 2 [file bmjopen-2025-102096.reviewer_comments.pdf]

PEER REVIEW HISTORY

BMJ Open publishes all reviews undertaken for accepted manuscripts. Reviewers are asked to complete a checklist review form and are provided with free text boxes to elaborate on their assessment. These free text comments are reproduced below.

ARTICLE DETAILS

Title (Provisional)

Quantifying cost and health-related quality of life outcomes in different multimorbidity trajectories: a systematic review protocol

Authors

Corcoran, Neave; Wilson, Naomi; Mackinnon, Shona; Blake, Anna; Newport, Heather; Lawless, Robert; Jani, Bhautesh; Mair, Frances; Taylor, Rod; McIntosh, Emma

---

VERSION 1 - REVIEW

**Reviewer**	**1**
**Name**	**Tran, Phuong Bich**
**Affiliation**	**University of Oxford, Nuffield Department of Primary Care Health Sciences**
**Date**	**06-May-2025**
**COI**	**None**

This manuscript presents a well-structured protocol for a systematic review on the cost and HRQoL impacts of multimorbidity trajectories—an area with growing relevance. The review is motivated and has the potential to inform future research and health and social care planning.

Below are some areas which could benefit from improvement.

Introduction

1. The focus on "trajectories" is introduced too late in the introduction, but also is still a bit unclear. Since this is the central concept of the review, it should be defined and foregrounded earlier.

2. In relation to costs, the authors should clarify how their proposed review offers added value compared to this existing review, which included both cross-sectional and longitudinal studies: https://bmcmedicine.biomedcentral.com/articles/10.1186/s12916-022-02427-9

What new insights or methodological advances will this review provide beyond what's already covered?

3. The introduction cites several relevant studies but doesn't clearly separate what's already known from what this review adds. The idea is there, but framing could be improved to strengthen the rationale of the review.

Methods

1. The term "trajectory" is used frequently but remains under-defined in operational terms. Please clarify whether the focus is on temporal progression (e.g. rate or sequence of condition onset) or the potential addition of new conditions over time (if so, how will these be handled analytically?) Focusing only on the number of comorbidities may offer limited added value in advancing understanding of multimorbidity 'trajectories'.

2. A clear definition of what constitutes a long-term condition (LTC) for inclusion is missing. Further, will closely related conditions such as coronary heart disease and peripheral artery disease be considered distinct LTCs or part of a single category (e.g. CVD)? Will high cholesterol be considered an LTC or risk factor? What about episodic conditions - will they be included? Given the variability in how conditions may be reported and classified across studies, further clarification is needed on how the review team will handle categorisation and standardisation of disease definitions during data extraction and synthesis.

3. The current search strategy appears to rely heavily on generic terms such as "multimorbidity" and "comorbidities." While this is appropriate for capturing broad literature, it risks excluding a substantial body of relevant studies that explore longitudinal impacts on cost and HRQoL among individuals with two or more specific conditions—without necessarily labelling them as "multimorbidity" studies. The authors should consider incorporating condition-pair or cluster terms, or at least test broader Boolean combinations involving common comorbidity groupings. Alternatively, the authors should clearly justify why such studies are intentionally excluded and acknowledge the potential impact of this decision on the scope and findings of the review.

4. This review requires a more rigorous quality assessment process, especially given its focus on two distinct outcomes: cost and HRQoL. Each outcome presents different methodological challenges, and depending on the study design of the individual studies, there are tailored checklists available (e.g., Larg & Moss (2011), Papaioannou et al. (2013), Xie et al. (2015)) that could be adapted alongside the JBI Checklist for Cohort Studies.

5. The authors should clarify how they'll handle heterogeneity in cost data—e.g. currency, price year, and cost perspective—and whether costs will be standardised.

6. The authors note early on that studies may be heterogeneous and that meta-analysis may not be appropriate. However, it would be helpful to clarify what types or thresholds of heterogeneity (e.g., clinical, methodological, statistical) would guide the decision.

7. Regarding the impact of PPI, please clarify how involvement specifically influenced the development of this protocol? (e.g., did it help shape the research questions, selection of outcomes, or review methodology?)

Discussion

1. The authors repeatedly state that the review will provide a “comprehensive summary,” yet they also acknowledge that existing evidence in this area may be sparse. As this is a protocol and the volume of available literature is not yet known, it would be more appropriate to use cautious language, such as “aims to provide an overview” or “will synthesise the available evidence.”

2. "Timely intervention is likely to be influenced by cost and HRQoL in relation to the cost-effectiveness of services and interventions…"

This is confusingly phrased: cost and HRQoL are outcomes used to assess cost-effectiveness, but they don’t usually influence whether an intervention is timely. Instead, what matters is whether interventions delivered at specific time points improve cost and HRQoL outcomes — not that cost and HRQoL influence the timing.

3. “Existing research on multimorbidity trajectories has highlighted considerable heterogeneity in methodologies used, including in study design, population(s) of interest, sampling strategies and analytic approaches…”

The paragraph identifies methodological heterogeneity, but doesn’t explain why it’s a problem. Does it limit comparability? Reduce generalisability?

4. “Findings from this work could inform health policy, allocation of healthcare resources and guide the design of future health services and interventions which address both cost-effectiveness, optimisation and sustainability concerns, but which are also patient-centred, optimising patient experience and minimising treatment burden, disruption and HRQoL impact for people with multimorbidity.”

There’s a mismatch between the data type and the claimed impact. Longitudinal economic and utility data help inform system-level efficiency questions. Patient-centredness and treatment burden require direct engagement with lived experience, which this review doesn’t aim to synthesise.

**Reviewer**	**2**
**Name**	**Lieber, Judith**
**Affiliation**	**London School of Hygiene and Tropical Medicine Faculty of Epidemiology and Public Health**
**Date**	**15-May-2025**
**COI**	**None**

Thank you for the opportunity to review this protocol. This is an important topic and a useful evidence synthesis. I have minor comments.

- The abstract seems to include a reference which isn't standard practice

- In the introduction and aims/objectives, sometimes HCRU is mentioned and other times it is not. It is clear from the methods section that HCRU outcomes will be assessed, so please consistently discuss HCRU alongside costs and HrQoL

- The strengths/limitations state the review will 'capture of all relevant evidence'. This review is limited to studies after 2010 in the English language, so I don't think this is true. Please soften this statement

- Some sentences in the background/literature review are very long which makes the text difficult to follow. I am also unclear why the Fu et al. study is described when the results are not informative for this research question

- The background mentions that a few systematic reviews have found an inverse relationship between MM and HrQoL. Please highlight how this review differs from these previous reviews, i.e., what will be novel?

- I am unclear what the following sentence means - 'Research in this area has highlighted significant heterogeneity in original studies comparing multimorbidity and HRQoL measures.' Studies that looked at the association between MM and HrQoL?

- The exposure is described a 'different MM trajectories over time'. I am not clear what 'different' means in this sentence. Can you also define what you mean by MM trajectory over time?

- Re the comparator, could one MM trajectory be compared against another MM trajectory? This isn't mentioned.

- The outcomes are described as 'over time'. Does this mean that all the analyses/outcomes will be change outcomes? I.e., difference in HrQoL between baseline (no MM) and follow-up (MM). That would exclude studies that compare cross-sectional HrQoL at follow-up between groups who developed MM and did not develop MM. You mention this type of study in the comparator section, but inversely do not mention comparing across time in the same person.

- Re the exposure, the protocol states that a study will be included if it 'uses a measure that explicitly quantifies multimorbidity'. Does this mean that only studies that 'self-define' as multimorbidity or comorbidity studies will be included? That would miss a lot of relevant evidence, e.g., studies may state they are examining metabolic syndrome (which is essentially a MM cluster)

- Please explain how you will define long-term condition. For example, are obesity and hypertension risk-factors or conditions?

- I am unclear whether the costs outcomes are always monetary. I.e., would a self-reported binary outcome of job loss or lost income be included?

- The background states that relatively few longitudinal studies of the impact of MM on costs/HrQoL have been done. If this is the case, I don't think the argument for only reviewing

papers from 2010 (or restricting to self-defined MM/CoM studies) is very strong. Please reconsider this or explain this rationale further if the review has already begun.

- In the study characteristics, you plan to collect proportion female at birth. I would assume that most cohort studies ask about gender rather than sex at birth. Could this be redefined as proportion female at birth / women?

- It would be useful to collect the covariates adjusted for in multivariate analyses

- I am unclear what 'main results' refers to in the data extraction table – the study conclusions? Please explain this further

- Please add a limitations section

- I am unclear if this review has already started. If so, please add the dates

---

VERSION 1 - AUTHOR RESPONSE

Reviewer: 1<br>***Dr. Phuong Bich Tran, University of Oxford***			
Introduction *The focus on "trajectories" is introduced too late in the introduction, but also is still a bit unclear. Since this is the central concept of the review, it should be defined and foregrounded earlier.*	*Many thanks for this valid point which we have endeavoured to address; the Introduction section has been revised, and the concept of trajectories is now introduced earlier (in paragraph 3) as suggested, and foregrounding of the concept has been strengthened in this same paragraph.*	*Page 3, section: Introduction, paragraph 3*	*'Little work to date has explored the extent and scale of longitudinal costs and impacts on HRQOL as individuals develop multimorbidity over time, progressing from having zero or one single LTC to MLTCs i.e. different multimorbidity trajectories. The Academy of Medical Sciences reports that 'longitudinal data on time trends in costs for the management of multimorbidity are required for a range of settings. Longitudinal data on multimorbidity trajectories may refer to e.g., timing or sequencing of LTC accumulation, longitudinal clustering of LTCs or key health-disease state transitions.'*
In relation to costs, the authors should clarify how their proposed review offers added	*Many thanks for this comment and we agree it is important to make*	*Pages 3-4, section: Introduction – whole section*	*'One common limitation of many studies considering the costs associated with multimorbidity is the scope of costs considered, where non-medical and*

value compared to this existing review, which included both cross-sectional and longitudinal studies: https://bmcmedicine.biomedcentral.com/articles/10.1186/s12916-022-02427-9 What new insights or methodological advances will this review provide beyond what's already covered?	*clear what evidence gaps this review aims to address. In relation to costs specifically, this review aims to focus specifically on longitudinal research on the association between different multimorbidity trajectories over time and HCRU/cost impacts, an area which has been highlighted by multiple sources as an evidence gap, with the potential to offer further insights into multimorbidity progression, drivers, associated impacts and interventions. This has been described in the reworked Introduction section, and cites the previous systematic review which you have kindly linked to, and which has been a valued key reference during this research.*	*has been revised, and drawing specific attention to the editing of paragraph 4, sentence 2, and the addition of new paragraph 5*	*indirect costs (e.g. work absence, loss of income) are often not included, potentially underestimating the true total societal costs and impacts of multimorbidity.'* <br>*'A 2022 systematic review by Tran et al exploring costs of multimorbidity detected certain time points or 'transition stages' in a health trajectory where costs concentrated: around the time of a new diagnosis and in the six months preceding death. The authors underscore the importance of further longitudinal multimorbidity costs research to better understand the dynamic nature of health states, disease trajectories and associated costs, and thereby target future interventions to best prevent multimorbidity progression.'*

The introduction cites several relevant studies but doesn't clearly separate what's already known from what this review adds. The idea is there, but framing could be improved to strengthen the rationale of the review.	*Thank you very much for this comment which has been very instructive for improving the Introduction. The Introduction section has been reworked in full with consideration given to logical ordering of paragraphs/sections to make the thread more coherent, framing the ideas in context, removing less relevant/critical sections and adding in new points and references relevant to the research questions.*	*Pages 3-4, section: Introduction*	
***Methods** The term "trajectory" is used frequently but remains under-defined in operational terms. Please clarify whether the focus is on temporal progression (e.g. rate or sequence of condition onset) or the potential addition of new conditions over time (if so, how will these be handled*	*Many thanks for this important point. As it is expected that there may be significant variability in how 'multimorbidity trajectories' are operationalised in different included studies this was left somewhat open-ended in the initial protocol*	*Page 6, section: Exposure, paragraphs 4-5*	*'It is anticipated that there will be substantial variety in the way that 'multimorbidity trajectories' is operationalised in studies included in the review. For example, approaches could focus on disease sequence i.e. the order in which LTCs develop in an individual; timing or rate of multimorbidity development e.g. including age at onset, longitudinal clusters or groupings of LTCs which tend to pattern together over time e.g. physical-mental health clusters or cardiometabolic clusters; disease state transitions; changes*

analytically?) Focusing only on the number of comorbidities may offer limited added value in advancing understanding of multimorbidity 'trajectories'.	*manuscript, however I fully understand it is important to explain this further and appreciate your comment. Additional detail relating to this point has been added in the Exposure section of Methods and Analysis*		*in simple LTC count or comorbidity index measure over time.*<br><br>*Depending on the variety of approaches taken to define multimorbidity trajectories found in include studies, data analysis and synthesis methods will be tailored accordingly, e.g. by grouping studies which employ similar methodologies for synthesis and presentation of results.'*
A clear definition of what constitutes a long-term condition (LTC) for inclusion is missing. Further, will closely related conditions such as coronary heart disease and peripheral artery disease be considered distinct LTCs or part of a single category (e.g. CVD)? Will high cholesterol be considered an LTC or risk factor? What about episodic conditions - will they be included? Given the variability in how conditions may be reported and classified across studies, further	*Thank you for this comment and I agree it is important to clarify this. An additional sentence has been added to further explain how varying definitions of LTCs (e.g. disease vs. risk factor) and of multimorbidity itself will be treated in the review.*	*Page 6, section: Exposure, paragraphs 2-3*	*'It is expected that there may be significant variability in the way multimorbidity is defined within included studies,(32) including in terms of which specific LTCs are included.(33) Based on existing evidence, the inclusion of diagnoses which may be variously considered as conditions or risk factors e.g. hypertension, hypercholesterolaemia, obesity, within different multimorbidity measures, is expected to vary.(34)*<br><br>*LTCs will be considered according to the definitions established by Barnett et al where closely related conditions e.g. coronary heart disease and peripheral artery disease are considered as distinct LTCs.(1) Obesity and high cholesterol will generally be regarded as risk factors rather than LTCs, and hypertension as a LTC, in accordance with Ho et al's 2022 Delphi study.(2) However, since it is expected that*

clarification is needed on how the review team will handle categorisation and standardisation of disease definitions during data extraction and synthesis.			*definitions of LTCs and lists of included LTCs will likely vary between included studies, provided that rationale for a given approach is demonstrated, studies will not be excluded on the basis of varying LTC inclusion, provided they meet the other review eligibility criteria.'*
The current search strategy appears to rely heavily on generic terms such as "multimorbidity" and "comorbidities." While this is appropriate for capturing broad literature, it risks excluding a substantial body of relevant studies that explore longitudinal impacts on cost and HRQoL among individuals with two or more specific conditions—without necessarily labelling them as "multimorbidity" studies. The authors should consider incorporating condition-pair or cluster terms, or at least test broader Boolean combinations involving common comorbidity groupings. Alternatively, the authors should clearly	*Thank you for this important point. Our intention is to focus solely on studies exploring the development of multimorbidity over time (and reporting associated HCRU, cost and HRQoL outcomes). According to our study design eligibility criteria, comorbidity studies e.g. those studying an index LTC and subsequent development of other specific LTCs, or those considering two specific conditions, are to be excluded* ***apart from*** *those where the longitudinal development of multimorbidity itself is reported, which would be*	*Page 8, section: Study design, paragraph 1*<br><br>*Pages 13-14, section: Discussion, paragraph 4, sentence 5.*	*'This review will focus on multimorbidity trajectories and therefore may exclude comorbidity studies of index conditions, where development of multimorbidity is not reported.'*

justify why such studies are intentionally excluded and acknowledge the potential impact of this decision on the scope and findings of the review.	*included. This is outlined in the section Study design.*<br><br>*Whilst we accept that this approach will exclude purely comorbidity studies (i.e. studies with an index condition or LTC pair which are not studying multimorbidity trajectories), this is aligned with the distinct definitions of multimorbidity and comorbidity as defined in the 2021 Journal of Multimorbidity and Comorbidity editorial (https://journals.sagepub.com/doi/full/10.1177/2633556521993993), and for the purposes of this review, the concept of interest is specifically multimorbidity development. This limitation to the scope of the review is now acknowledged in the Discussion*		

	section as indicated.		
This review requires a more rigorous quality assessment process, especially given its focus on two distinct outcomes: cost and HRQoL. Each outcome presents different methodological challenges, and depending on the study design of the individual studies, there are tailored checklists available (e.g., Larg & Moss (2011), Papaioannou et al. (2013), Xie et al. (2015)) that could be adapted alongside the JBI Checklist for Cohort Studies.	*Thank you for these very useful references and suggestions. Adaptations have been made to the quality assessment/risk of bias tool used in the review based on the recommended Papaioannou quality assessment guidance, and a checklist for critical appraisal of cost of illness studies by Schnitzler et al that linked from the Larg & Moss guidance – many thanks indeed for these useful suggestions which will undoubtedly enhance the review.*	*Page 11, section: Quality assessment, paragraph 1, sentence 2.*<br><br>*Page 11, Box 2. Quality assessment domains, section: 5 b), c)*	*'Further quality appraisal elements specific to cost of illness (COI) studies and health state utility value (HSUV) studies have also been incorporated into the tool.'*<br><br>*'b) Where HCRU/cost outcomes are reported, are all included components of resource use identified, measured and valued in monetary terms? Are future costs discounted? Are results presented by cost category/sector?*<br><br>*c) Where HRQoL outcomes are reported, is the measure used valid in the population studied?'*
The authors should clarify how they'll handle heterogeneity in cost data—e.g. currency, price year, and cost perspective—and whether costs will be standardised.	*Many thanks for pointing this out and apologies for the omission. These aspects of cost data will be recorded during data extraction and costs will be standardised where possible using*	*Page 7, Table 2. Outcomes of interest, section: Healthcare resource use/Cost*<br><br>*and*	*'Perspective*<br><br>*Currency*<br><br>*Unit cost source(s)*<br><br>*Base year for costs*<br><br>*Discounting applied Y/N and if Y, discount rate*<br><br>*Full economic evaluation Y/N and if Y, economic evaluation framework: cost-*

	appropriate year and currency, applying Purchasing Power Parity rates. The relevant cost data elements have been added to Table 2. Outcomes of interest, and Table 3. Data extraction, along with other relevant cost data which is being collected. We hope this helps clarify.	*Page 10, Table 3. Data extraction, section: HCRU/Cost outcomes*	*effectiveness analysis, cost-utility analysis, cost-benefit analysis, cost-consequence analysis'*
The authors note early on that studies may be heterogeneous and that meta-analysis may not be appropriate. However, it would be helpful to clarify what types or thresholds of heterogeneity (e.g., clinical, methodological, statistical) would guide the decision.	*Many thanks for this comment and suggestion which has improved the clarity of the explanation of our approach to data synthesis in this review. Further detail has been added to the Data synthesis section which I hope addresses this point.*	*Page 12, section Data synthesis, paragraph 1, sentences 2-5*	*'Initial scoping of the literature suggests there may be limited existing published evidence addressing our stated research questions, and that significant methodological heterogeneity may be found between studies, including in terms of clinical focus, study designs, populations and outcomes of interest, effect measures and statistical methodologies. For these reasons, meta-analysis may not be possible, therefore an approach such as Synthesis Without Meta-analysis (SWiM) may be most appropriate.*<br><br>*The SWiM guidelines acknowledge that formal statistical investigation of heterogeneity may not always be possible or appropriate, and offer alternative approaches to examine heterogeneity such as ordering tables and/or figures by study characteristics e.g. study design, population, or outcomes.'*

Regarding the impact of PPI, please clarify how involvement specifically influenced the development of this protocol? (e.g., did it help shape the research questions, selection of outcomes, or review methodology?)	*Thank for this helpful comment. The section outlining PPI engagement and contribution has been expanded to specifically describe the PPI contribution in developing the review protocol.*	*Page 12, section: Methods > Patient and Public Involvement. Paragraph 1, sentence 3*	*'PPI engagement contributed particularly to development of the research questions, the selection of relevant outcomes, particularly inclusion of indirect costs, and the use of a double-stranded search strategy encompassing both HCRU/cost and HRQoL outcomes.'*
Discussion *The authors repeatedly state that the review will provide a "comprehensive summary," yet they also acknowledge that existing evidence in this area may be sparse. As this is a protocol and the volume of available literature is not yet known, it would be more appropriate to use cautious language, such as "aims to provide an overview" or "will synthesise the available evidence."*	*Thanks for this very valid point and the statements have been softened accordingly, in line with your helpful suggestions to demonstrate a more cautious approach.*	*Page 2, section: Abstract > Introduction, paragraph 2*<br><br>*Page 4, section: Introduction, paragraph 11*<br><br>*Page 13, section: Discussion, paragraph 1*	*'This systematic review will synthesise the available evidence on HCRU, costs and HRQoL impacts of different multimorbidity trajectories.'*<br><br>*'This systematic review will for the first time synthesise the available evidence on HCRU, cost and HRQoL impacts of different trajectories of multimorbidity development.'*<br><br>*'This systematic review will synthesise the available evidence on HCRU and costs, and HRQoL impacts of different longitudinal trajectories of multimorbidity development.'*

		Page 14, section: Discussion, paragraph 6	*'This work is novel, as to date, there is no systematic overview of literature relating to HCRU, cost and HRQoL impacts of different multimorbidity trajectories.'*
"Timely intervention is likely to be influenced by cost and HRQoL in relation to the cost-effectiveness of services and interventions…"<br>*This is confusingly phrased: cost and HRQoL are outcomes used to assess cost-effectiveness, but they don't usually influence whether an intervention is timely. Instead, what matters is whether interventions delivered at specific time points improve cost and HRQoL outcomes — not that cost and HRQoL influence the timing.*	*Thank you, yes, we appreciate this wording is confusing and the point may not be entirely clear to the reader so thank you for highlighting this. The sentence has been reworded to clarify the point.*	*Page 13, section: Discussion, paragraph 2, sentence 3*	*The timing of interventions is likely to influence costs and HRQoL trajectories and hence longitudinal information on HCRU, costs and HRQoL is needed.*
"Existing research on multimorbidity trajectories has highlighted considerable heterogeneity in methodologies used, including in study design, population(s) of interest, sampling	*Thank you for this comment; further explanation has been added that this anticipated heterogeneity in studies may limit comparability and generalisability.*	*Page 13, section: Discussion, paragraph 3, sentence 2*	*'Existing research on multimorbidity trajectories has highlighted considerable heterogeneity in methodologies used, including in study design, population(s) of interest, sampling strategies and analytic approaches, which may limit the comparability of studies included in this review, as well as wider generalisability of findings.'*

strategies and analytic approaches…"<br><br>*The paragraph identifies methodological heterogeneity, but doesn't explain why it's a problem. Does it limit comparability? Reduce generalisability?*			
"Findings from this work could inform health policy, allocation of healthcare resources and guide the design of future health services and interventions which address both cost-effectiveness, optimisation and sustainability concerns, but which are also patient-centred, optimising patient experience and minimising treatment burden, disruption and HRQoL impact for people with multimorbidity."<br><br>*There's a mismatch between the data type and the claimed impact. Longitudinal economic and utility data help inform system-level efficiency questions. Patient-*	*Many thanks for this useful comment and I appreciate your insight here. The statement you have quoted has been amended accordingly to present a more accurate reflection of potential impacts of the data types studied in this review.*	*Page 14, section: Discussion, paragraph 8*	*'Findings from this work could inform health policy, allocation of healthcare resources and guide the design of future health services and interventions which address both cost-effectiveness, optimisation and sustainability concerns, whilst mitigating HRQoL detriment for people with multimorbidity.'*

centredness and treatment burden require direct engagement with lived experience, which this review doesn't aim to synthesise.			
Reviewer: 2 Dr. Judith Lieber, London School of Hygiene and Tropical Medicine Faculty of Epidemiology and Public Health			
The abstract seems to include a reference which isn't standard practice	*Many thanks for pointing this out and we appreciate this is not standard practice in an abstract. The reference has been removed. (The relevant reporting statement is still referenced in the main text under Data synthesis)*		
In the introduction and aims/objectives, sometimes HCRU is mentioned and other times it is not. It is clear from the methods section that HCRU outcomes will be assessed, so please consistently discuss HCRU alongside costs and HrQoL	*Thanks for this helpful comment; consistent reference to HCRU alongside costs and HRQoL outcomes has been added throughout the text for clarity and consistency.*	*Multiple points throughout the manuscript, can be seen highlighted in red in the tracked changes version.*	

The strengths/limitations state the review will 'capture of all relevant evidence'. This review is limited to studies after 2010 in the English language, so I don't think this is true. Please soften this statement	*Thank you for this valid comment, the sentence has been softened as suggested.*	*Page 3, Box 1, bullet point 1*	*'Inclusion of a broad range of multimorbidity, cost and health-related quality of life outcome measures as described here in the review studies will enable systematic capture of relevant evidence'*
Some sentences in the background/literature review are very long which makes the text difficult to follow. I am also unclear why the Fu et al. study is described when the results are not informative for this research question	*Many thanks for these helpful and instructive comments. The Introduction section has been reworked in full, making sentences more concise and removing extraneous detail/reference, for example the Fu reference which as per your comment, added little to the thread of the Introduction overall.*	*Pages 3-4, section: Introduction*	
The background mentions that a few systematic reviews have found an inverse relationship between MM and HrQoL. Please highlight how this	*Thank you for this feedback, I agree it is important to make clear the potential novel findings proposed by this review, and*	*Page 4, section: Introduction, paragraph 6, sentences 2-3*	*'There is growing cross-sectional evidence on the inverse relationship between greater long-term condition counts and HRQoL outcomes across multiple systematic reviews. However, key reviews of the association between multimorbidity and HRQoL outcomes have identified a*

review differs from these previous reviews, i.e., what will be novel?	*the evidence gap it aims to fill in relation to HRQoL impacts of multimorbidity trajectories. Apologies this was not made clearer in the initial submission. Further detail on this has been added in the revised Introduction section as pasted here.*<br><br>*(N.B. Additional detail has also been added on the potential novel findings of this review in relation to cost outcomes in Introduction, paragraph 5, sentence 2)*		*sparsity of longitudinal studies and suggest that longitudinal research is required to aid understanding of the impacts of change in extent of multimorbidity over time on HRQoL.'*
- I am unclear what the following sentence means - 'Research in this area has highlighted significant heterogeneity in original studies comparing multimorbidity and HRQoL measures.' Studies that looked at the association between MM and	*Many thanks for this comment and we appreciate this sentence was unclear. It has been reworded for clarity, in line with your question/suggestion.*	*Page 4, section: Introduction, paragraph 6, sentence 4*	*'Research in this area has highlighted significant heterogeneity in studies exploring associations between multimorbidity and HRQoL outcomes.'*

HrQoL?			
The exposure is described a 'different MM trajectories over time'. I am not clear what 'different' means in this sentence. Can you also define what you mean by MM trajectory over time?	*Thank you for your helpful comment. The use of 'different' here is meant to allow for variation in several aspects of how multimorbidity trajectories develop longitudinally (or how they are defined or measured in a study), encompassing e.g. aspects such as rate of LTC accumulation, differences in sequencing of LTCs, specific longitudinal clusters/patterns of LTCs, or different disease state transitions (e.g. single LTC/multimorbidity /complex multimorbidity) etc.*<br><br>*Thank you also for querying clarity on definition of multimorbidity trajectories over time as this is an important point which was*	*Page 6, section: Exposure, paragraph 4*	*'It is anticipated that there will be substantial variety in the way that 'multimorbidity trajectories' is operationalised in studies. For example, approaches could focus on disease sequence i.e. the order in which LTCs develop in an individual; timing or rate of multimorbidity development e.g. including age at onset, longitudinal clusters or groupings of LTCs which tend to pattern together over time e.g. physical-mental health clusters or cardiometabolic clusters; disease state transitions; changes in simple LTC count or comorbidity index measure over time.'*

	underdeveloped in the original manuscript. It is expected that there may be significant variability in how 'multimorbidity trajectories' are operationalised in different included studies, therefore this was left somewhat open-ended in the initial protocol manuscript, however I understand it is important to define this further and am grateful for your question.<br><br>*Additional detail addressing both of these points has been added in the Exposure section of Methods and Analysis*		
Re the comparator, could one MM trajectory be compared against another MM trajectory? This isn't mentioned.	*Thank you for this comment and apologies that this was not made clearer. Yes, one multimorbidity trajectory could be compared to another*	*Page 7, section: Comparator, paragraph 3*	*'Participants who develop multimorbidity may be compared with adults who do not develop multimorbidity during the follow up period, i.e. those with zero or one long-term condition throughout follow-up, or may compare effect size of different multimorbidity trajectories on HCRU/cost or HRQoL outcomes against each other,*

	multimorbidity trajectory and this is stated on page 6, in section Methods and Analysis, subsection Comparator, paragraph 3: 'Participants who develop multimorbidity may be compared with adults who do not develop multimorbidity during the follow up period, i.e. those with zero or one long-term condition throughout follow up, ***or may compare effect size of different multimorbidity trajectories on HCRU/cost or HRQoL outcomes against each other****, although studies will not be excluded if there is no comparator.'*<br><br>*However, I agree this was not made clear in Table 1. Review inclusion criteria, section: Comparator, and so an additional*	*Page 5, Table 1. Review inclusion criteria, section: Comparator*	*although studies will not be excluded if there is no comparator.'*<br><br>*'Comparator may be adults with zero or one long-term condition, or comparing one multimorbidity trajectory with another; however studies that do not include a comparator or control group will not be excluded.'*

	clause has been added to this section to clarify this – many thanks for highlighting this omission.		
The outcomes are described as 'over time'. Does this mean that all the analyses/outcomes will be change outcomes? I.e., difference in HrQoL between baseline (no MM) and follow-up (MM). That would exclude studies that compare cross-sectional HrQoL at follow-up between groups who developed MM and did not develop MM. You mention this type of study in the comparator section, but inversely do not mention comparing across time in the same person.	*Thank you very much for this valid point and I appreciate these details need to be made clearer in the text. The longitudinal/'over time' element is the development/change in multimorbidity over time, and outcomes including HCRU, cost and/or HRQoL may be either cross-sectionally or longitudinally reported. So as per your comment, studies comparing cross-sectional HRQoL at follow-up could be included provided a longitudinal or 'trajectory' measure of multimorbidity development/change has been reported. I hope*	*Page 5, Table 1. Review inclusion criteria, section: Outcomes*	*'Measure of healthcare resource use and/or direct and indirect costs reported cross-sectionally or longitudinally*<br><br>*Measure of health-related quality of life outcomes reported cross-sectionally or longitudinally'*

	this makes sense and I have clarified the Outcomes section in Table 1. Review inclusion criteria, which I agree was not clear.		
Re the exposure, the protocol states that a study will be included if it 'uses a measure that explicitly quantifies multimorbidity'. Does this mean that only studies that 'self-define' as multimorbidity or comorbidity studies will be included? That would miss a lot of relevant evidence, e.g., studies may state they are examining metabolic syndrome (which is essentially a MM cluster)	*Thank you for this important point. Our intention is to focus solely on studies explicitly exploring the development of multimorbidity over time (and reporting associated HCRU, cost and HRQoL outcomes). According to our study design eligibility criteria, comorbidity studies e.g. those studying an index LTC and subsequent development of other specific LTCs, or those considering a specific LTC cluster e.g. metabolic syndrome, as opposed to multimorbidity development more broadly, are to be excluded* ***apart from*** *those where*	*Page 8, section: Study design, paragraph 1, sentences 2 and 3*<br><br>*Pages 13-14, section: Discussion, paragraph 4, sentence 5*	*'This review will focus on multimorbidity trajectories and therefore may exclude comorbidity studies of index conditions, where development of multimorbidity is not reported.'*

	the longitudinal development of multimorbidity itself is reported, which would be included. This is outlined in the section Study design.<br><br>*Whilst we accept that this approach will exclude purely comorbidity studies (i.e. studies with an index condition or LTC pair which are not studying multimorbidity trajectories), this is aligned with the distinct definitions of multimorbidity and comorbidity as defined in the 2021 Journal of Multimorbidity and Comorbidity editorial (https://journals.sagepub.com/doi/full/10.1177/26335565521993993), and for the purposes of this review, the concept of interest is specifically multimorbidity development. This limitation to the*		

	scope of the review is now acknowledged in the Discussion section as indicated.		
Please explain how you will define long-term condition. For example, are obesity and hypertension risk-factors or conditions?	*Thank you for this comment and I agree it is important to clarify this. An additional sentence has been added to further explain how different definitions of LTCs and of multimorbidity itself will be treated in the review.*	*Page 6, section: Exposure, paragraphs 2-3*	*'It is expected that there may be significant variability in definitions of multimorbidity operationalised by included studies,(32) including in terms of which specific LTCs are included in each definition.(33) Furthermore, based on existing evidence, the inclusion of diagnoses which may be variously considered as conditions or risk factors e.g. hypertension, hypercholesterolaemia, obesity, within different multimorbidity measures, is expected to vary.(34)*<br><br>*LTCs will be considered according to the definitions established by Barnett et al where closely related conditions e.g. coronary heart disease and peripheral artery disease are considered as distinct LTCs.(1) Obesity and high cholesterol will generally be regarded as risk factors rather than LTCs, and hypertension as a LTC, in accordance with Ho et al's 2022 Delphi study.(2) However, since it is expected that definitions of LTCs and lists of included LTCs will likely vary between included studies, provided that rationale for a given approach is demonstrated, studies will not be excluded on the basis of varying LTC inclusion, provided they meet the other review eligibility criteria.'*

I am unclear whether the costs outcomes are always monetary. I.e., would a self-reported binary outcome of job loss or lost income be included?	*Thank you for this query and apologies this was not clear. HCRU/cost outcomes for this review could include both monetary and non-monetary direct and indirect costs. Possible cost outcomes that may be included are detailed in Table 2. Outcomes of interest, and, as you suggested, may include loss of income, job loss or other outcomes e.g. reduced productivity, loss of benefits.*	*Page 7, section Outcomes, Table 2. Outcomes of interest, section: Healthcare resource use/Cost*	*'Indirect costs*<br>*Non-medical costs (transport, meals, accommodation etc)*<br>*Loss of income*<br>*Job loss*<br>*Reduced productivity*<br>*Loss of benefits*<br>*Opportunity costs'*
The background states that relatively few longitudinal studies of the impact of MM on costs/HrQoL have been done. If this is the case, I don't think the argument for only reviewing papers from 2010 (or restricting to self-defined MM/CoM studies) is very strong.	*Thank you for this comment. The choice of 2010 as an earlier date limit is based upon research from 2013 developing the definition of multimorbidity as conceptualised in this review (Le Reste JY, Nabbe P,*	*Page 8, section: Study identification > Electronic searches, paragraph 2, sentence 2*<br><br>*Page15, section:*	*'The start date of 2010 was chosen as the concept of multimorbidity as referred to in this review was defined and developed from the early 2010s.'*

Please reconsider this or explain this rationale further if the review has already begun.	*Manceau B, Lygidakis C, Doerr C, Lingner H, et al. The European General Practice Research Network presents a comprehensive definition of multimorbidity in family medicine and long term care, following a systematic review of relevant literature. J Am Med Dir Assoc. 2013;14(5):319-25.) therefore little relevant evidence would be expected to have been published prior to 2010. The review is already underway as per the Prospero registration (CRD42024537258) and so this valid point is now acknowledged as a limitation in the Discussion.*	*Discussion, paragraph 4, sentences 4-5*	*'2010 has been chosen as an earlier date search limit, based upon research from 2013 developing the definition of multimorbidity as conceptualised in this review, therefore little relevant evidence would be expected to have been published prior to 2010. However it is possible that this approach may exclude relevant studies published before 2010 and this is acknowledged as a potential limitation.'*
In the study characteristics, you plan to collect proportion female at birth. I would assume that most cohort studies ask about	*Thanks for this helpful suggestion. Prompted by this, I have done some wider research and consulted with my supervisory team.*	*Page 9, Table 3. Data extraction, section: Study characteristics*	*'Sex: Proportion female'*

gender rather than sex at birth. Could this be redefined as proportion female at birth / women?	*More often the biological characteristic 'sex' is reported, whereas 'gender' encompasses psychosocial and cultural factors and may or may not be concordant with sex (References: **JAMA**: https://jamanetwork.com/journals/jama/fullarticle/2577142#:~:text=Sex%20is%20recognized%20implicitly%20as,clayton@nih.gov).&text=Conflict%20of%20Interest%20Disclosures:%20All,Interest%20and%20none%20were%20reported. , **Nature/European Commission**: https://www.nature.com/articles/d41586-020-03459-y , **UK Biobank**: https://biobank.ctsu.ox.ac.uk/ukb/field.cgi?id=31 , https://pmc.ncbi.nlm.nih.gov/articles/PMC10161036/#:~:text=Significance,in%20clinical%20and%20survey%20research. , **UKRI/MRC**: https://www.ukri.or*		

	g/councils/mrc/guidance-for-applicants/policies-and-guidance-for-researchers/sex-in-experimental-design/) *This information has been amended in the Study characteristics section of Table 3. Data extraction.*		
It would be useful to collect the covariates adjusted for in multivariate analyses	*Many thanks for this very useful suggestion, that is really relevant information to include in the data extraction and the data extraction form has been updated accordingly. Detail has been added to Table 3. Data extraction noting this addition.*	*Page 9, Table 3. Data extraction, section: Study characteristics*	*'Covariates adjusted for in multivariate analysis'*
I am unclear what 'main results' refers to in the data extraction table – the study conclusions? Please explain this further	*Thank you for the comment and apologies, I can understand why this is unclear. This has been reworded to 'study key findings/conclusions'*	*Page 10, Table 3. Data extraction, section: Results, conclusions and quality*	*'Study key findings/conclusions'*

Please add a limitations section	*Many thanks for this comment. Limitations are included in Box 1. Strengths and limitations of the study; this presentation of the limitations is the format that has been recommended by the journal. Further limitations are also discussed in the section Discussion, paragraphs 4 and 5, and with new limitations added here in response to your helpful comments.*	*Page 3, Box 1. Strengths and limitations of the study*<br><br>*Page 13, section: Discussion, paragraph 4, sentences 4-5, paragraph 5, sentence 3*	*'• Meta-analysis may or may not be possible depending on quality of reporting and heterogeneity in included studies*<br>*• Existing evidence in this area may be sparse'*<br><br>*'However, it is possible that this approach may exclude relevant studies published before 2010 and this is acknowledged as a potential limitation. This review will focus on multimorbidity trajectories and therefore may exclude comorbidity studies of index conditions, where development of multimorbidity is not reported.*<br><br>*Should the included studies not be amenable to meta-analysis, this will be recognised as a limitation of the review, and SWiM methodology will provide an alternative systematic, transparent and rigorous synthesis approach.'*
I am unclear if this review has already started. If so, please add the dates	*Thank you for this comment/question. The review has started, with dates stated on page 1*	*Page 1, section: Protocol dates*	***'Protocol dates**: Feb 2024 – ongoing'*

VERSION 2 - REVIEW

Reviewer 1

Name Tran, Phuong Bich

Affiliation University of Oxford, Nuffield Department of Primary Care Health Sciences

Date 17-Jul-2025

COI

Thanks for the response and revision from the authors. I have no further comments.

Reviewer 2

Name Lieber, Judith

Affiliation London School of Hygiene and Tropical Medicine Faculty of Epidemiology and Public Health

Date 31-Jul-2025

COI

Thank you for responding to and dealing with my feedback.
